# METTL16 promotes MASLD progression through the regulation of lipid synthesis and immune response

**DOI:** 10.1186/s13062-026-00760-0

**Published:** 2026-04-09

**Authors:** Guixin Li, Long Wang, Li Zhang, Jinghui Guo, Qun Sun

**Affiliations:** 1 Department of Gastroenterology, Shanghai Sixth People’s Hospital Affiliated to Shanghai Jiao Tong University School of Medicine, Shanghai, 200233 China; 2 Department of Pathology, Shanghai Sixth People’s Hospital Affiliated to Shanghai Jiao Tong University School of Medicine, Shanghai, 200233 China

**Keywords:** Metabolic dysfunction-associated steatotic liver disease, RNA methylation, Methyltransferase-like 16, Cell death-inducing DFF45 like effector family members A, Immune response

## Abstract

**Background:**

Methyltransferase-like 16 (METTL16) is a recently identified m6A RNA methyltransferase. Although its role in hepatocellular carcinoma has been explored, its function in metabolic dysfunction-associated steatotic liver disease (MASLD) remains elusive.

**Methods:**

Expression profiles of methylation-related genes were analyzed in a cohort comprising 206 MASLD patients and 10 healthy controls. Functional assays were conducted using MASLD cell models and diet-induced mouse models. Bioinformatic analyses were performed to evaluate immune cell infiltration and signaling pathway activation. Potential transcriptional regulators and small-molecule inhibitors of METTL16 were predicted through multiple databases.

**Results:**

METTL16 expression was markedly upregulated in MASLD tissues and closely correlated with lipid metabolism-related pathways. Knockdown of METTL16 alleviated hepatic steatosis, insulin resistance, and fibrosis in high-fat diet-fed mice. Mechanistically, cell death-inducing DFF45-like effector family members A (CIDEA) was identified as a downstream mediator of METTL16-driven hepatic steatosis. Elevated hepatic METTL16 expression was associated with increased infiltration of innate immune cells, activation of immune-related signaling pathways, and upregulated expression of pro-fibrotic genes. Database mining further revealed that METTL16 may regulate immune-associated genes at both transcriptional and post-transcriptional levels. Additionally, EGR1 and MYC were identified as potential upstream transcriptional regulators of METTL16, and eleven small molecules were predicted to bind to and inhibit its biological activity.

**Conclusion:**

METTL16 promotes hepatic steatosis and immune-mediated fibrogenesis in MASLD. Targeting the METTL16-CIDEA axis or inhibiting METTL16 activity may serve as a promising therapeutic strategy for the treatment of MASLD.

**Supplementary Information:**

The online version contains supplementary material available at 10.1186/s13062-026-00760-0.

## Introduction

Metabolic dysfunction-associated steatotic liver disease (MASLD), previously termed nonalcoholic fatty liver disease, has a global prevalence of approximately 30% [1]. Metabolic dysfunction-associated steatohepatitis (MASH), the progressive form of MASLD, is characterized by hepatic steatosis, lobular inflammation, and hepatocyte injury, with or without fibrosis [2, 3]. Despite substantial progress in related research, the molecular mechanisms governing lipotoxicity and inflammation in MASH remain not fully elucidated.

N^6^-methyladenosine (m^6^A) is the most abundant post-transcriptional modification in eukaryotic mRNA, accounting for nearly half of all methylated ribonucleosides [4]. This dynamic and reversible modification is orchestrated by m^6^A methyltransferases (referred to as writers), demethylases (erasers), and RNA-binding proteins (readers) [4]. Accumulating evidence demonstrates that m^6^A modulates RNA translation, degradation, folding, splicing, and transport, thereby regulating RNA stability and gene expression in the context of MASLD. In animal models of MASLD, hypermethylated genes primarily impact lipid metabolism [5, 6]. In patients with MASLD, compared to healthy controls, the expression levels of methyltransferase-like 3 (METTL3), METTL14, and fat mass and obesity-associated protein (FTO) are upregulated in the liver, whereas those of Wilms tumor 1-associated protein (WTAP), RNA-binding motif protein 15 (RBM15), YT521-B homology domain-containing 1 (YTHDC1), YTHDC2, insulin-like growth factor 2 mRNA binding protein 1 (IGF2BP1), heterogeneous nuclear ribonucleoprotein C (HNRNPC), and HNRNPA2B1 are downregulated [7].

m^6^A regulators exert complex, context-dependent effects on hepatic lipid homeostasis in MASLD. Among m^6^A writers, METTL13 inhibits autophagosome-lysosome fusion [8] and drives the progression from simple steatosis to steatohepatitis and fibrosis via CD36, CCL2, and TGF-β1 [9, 10]. However, it can also suppress de novo lipogenesis and inflammation through FASN and DDIT4 [11, 12]. METTL14 promotes MASLD progression by modulating ACLY and SCD1, while its regulation of NLRP3 can restore impaired hepatic insulin sensitivity [13, 14]. Regarding m^6^A erasers, FTO enhances lipid accumulation via SREBP1c, ChREBP, PPARγ, and CEBPα [15, 16]. As for readers, IGF2BP2 induces hepatic steatosis via the PTEN and AKT pathways, whereas YTHDC2 counteracts diet-induced steatosis by regulating multiple lipid synthesis genes [17, 18].

Activation of immune-inflammatory responses, in which m^6^A RNA modification acts as an epigenetic regulator, plays a pivotal role in the onset and progression of MASLD. For instance, METTL3-mediated methylation of STAT1 mRNA promotes M1 macrophage polarization, whereas knockdown of METTL3 enhances M2 polarization [19]. In addition, METTL3-dependent RNA methylation facilitates the activation and function of dendritic cells [20]. Moreover, deficiency of METTL3 disrupts T-cell differentiation and homeostasis [21], and specific deletion of METTL3 in regulatory T cells impairs their suppressive activity [22]. Likewise, deletion of METTL14 blocks B-cell maturation and impairs IL-7-induced pre-B cell proliferation [23]. However, the role of m^6^A methylation in immune regulation during MASLD progression remains largely unexplored. A recent study reported that the downregulation of METTL14 drives the differentiation of S100A4⁺ monocyte-derived macrophages, thereby accelerating MASLD progression [24].

In our previous study, we demonstrated that METTL16, a novel methyltransferase, was significantly upregulated in both in vivo and in vitro MASLD models. Additionally, we identified the cell death-inducing DFF45 like effector family members A (CIDEA) as a key gene driving MASLD progression [25]. These findings imply a potential association between METTL16-mediated m^6^A modification and the regulation of lipogenic pathways in MASLD. However, the precise molecular mechanisms through which METTL16 modulates MASLD, particularly its regulatory interplay with CIDEA, remain elusive.

In the present study, we systematically investigated the role of METTL16 in MASLD, with a focus on its effects on hepatic steatosis, insulin resistance, immune responses, and fibrosis, as well as its regulatory role in CIDEA expression. This work aims to provide a foundation for the development of METTL16-targeted therapeutic strategies for MASLD.

## Methods and materials

### Plasmid and shRNA construction

The CIDEA expression plasmid (p-CIDEA) was generated by inserting the full-length CIDEA coding sequence into the pcDNA3.1 vector (Asia-Vector Biotechnology, China). Gene-specific primers (CIDEA-F: 5’-CTCGGATCCGCCACCATGGAGGCCGCCCGGGAC  -3’; CIDEA-R: 5’-CCCTCTAGACTCGAGTCCACACGTGAACCTGCCCT - 3’) were used to amplify the CIDEA gene via PCR. The pcDNA3.1 vector was linearized using restriction enzymes XhoI and BamHI (New England Biolabs, USA). Subsequently, the amplified CIDEA fragment and linearized vector were ligated through a ligation-independent cloning approach, which was facilitated by Exonuclease III (BioFriend, China). Following transformation into E. coli DH5α competent cells, positive clones were selected based on antibiotic resistance and validated by colony PCR and Sanger sequencing to confirm the correct insertion of the CIDEA gene and the integrity of its sequence.

The METTL16 expression plasmid (p-METTL16) was constructed as described previously [25]. shRNA targeting METTL16 and CIDEA were purchased from Asia-Vector Biotechnology (China).

### Cell transfection and treatment

The human hepatoma cell line HepG2 (American Type Culture Collection, USA) was maintained in Dulbecco’s Modified Eagle’s Medium (DMEM, BasalMedia, China) supplemented with 10% fetal bovine serum (NEWZERUM, New Zealand), 100 IU/mL penicillin, and 100 µg/mL streptomycin (Sangon Biotech, China). HepG2 cells were seeded onto rat tail collagen I-coated plates and allowed to adhere for 8 h. Following cell attachment, the cells were transfected with one of the following constructs: p-METTL16 (METTL16-OE), METTL16 shRNA (shMETTL16), p-CIDEA (CIDEA-OE), CIDEA shRNA (shCIDEA), p-METTL16 combined with CIDEA shRNA (METTL16-OE+shCIDEA), or METTL16 shRNA combined with p-CIDEA (shMETTL16 + CIDEA-OE). To establish the in vitro MASLD model, 24 h after transfection, the cells were treated with 300 µM palmitic acid (PA) for an additional 24 h. Cell supernatants and lysates were then collected for subsequent experimental analysis.

Additionally, HepG2 cells with METTL16 overexpression were cultured for 24 h, followed by treatment with 5 µg/mL actinomycin D to block transcription for 0, 2, 4, 6, and 8 h. RNA was then harvested for downstream analysis.

### Human liver specimens and animal models

Human liver specimens were obtained from the para-carcinoma tissues of patients who underwent surgical resection for hepatocellular carcinoma. According to the degree of fatty infiltration in the adjacent liver tissue, the samples were stratified into two groups: a control group with mild or absent steatosis, and a hepatic steatosis group. The use of human liver specimens in this study was approved by the Ethics Committee of Shanghai Sixth People’s Hospital (Approval No. 2020-069). Written informed consent was obtained from all patients prior to sample collection.

Six-week-old male specific pathogen-free C57BL/6J mice were obtained from Beijing Vital River Laboratory Animal Technology Co., Ltd. (VRL, China). After a two-week acclimatization period under standard conditions, the mice were randomly divided into three experimental groups (*n* = 5 per group): (1) Control group: mice were fed a standard chow diet; (2) MASLD group: mice were fed a high-fat (HF) diet (catalog No.D12492, VRL, China); (3) MASLD+shMettl16 group: mice were fed HF diet and received intravenous injections of adeno-associated virus (AAV) harboring shMettl16 (dosage: 1 × 10^12^ viral genomes per mouse). The HF diet intervention was maintained for 16 weeks.

Mice were housed in a controlled environment with a 12 h light/dark cycle, with ad libitum access to food and water throughout the study. At the end of the experimental period, following a 12 h overnight fast, mice were euthanized. Sera and liver tissues were immediately harvested and stored at -80 °C for further analysis. All experimental procedures were conducted in accordance with the guidelines approved by the Institutional Animal Care and Ethics Committee (Approval No. AD2022189).

### Biochemical analyses, glucose tolerance test (GTT) and insulin tolerance test (ITT)

Triglyceride (TG) and total cholesterol (TCHO) levels in HepG2 cells and mouse serum were determined using commercial assay kits (Asia-Vector Biotechnology, China) in strict accordance with the manufacturer’s protocol. Serum insulin levels were quantified by enzyme-linked immunosorbent assay (ELISA). For the GTT, mice were fasted for 12 h, followed by intraperitoneal injection of glucose at a dose of 2 g/kg body weight. For the ITT, mice were fasted for 12 h before receiving an intraperitoneal injection of insulin at 0.75 U/kg body weight. Blood glucose levels were measured via tail vein sampling at 0, 30, 60, 90, and 120 min post-injection to evaluate glucose homeostasis and insulin sensitivity.

### Hematoxylin & eosin (H&E), Masson, Oil red O, and immunohistochemical staining

Mouse liver tissues were initially fixed in 4% paraformaldehyde for 24 h, and subsequently embedding in paraffin. The resulting paraffin sections were subjected to histological staining, including H&E staining for observation of general morphological features, and Masson’s trichrome staining for assessment of hepatic fibrosis.

Lipid accumulation was evaluated by fixing both frozen liver tissues and cultured cells. Frozen liver tissues were fixed with a standard fixative, whereas cultured cells were fixed in 4% paraformaldehyde. After fixation, samples were rinsed and equilibrated in 60% isopropanol. Lipid staining was conducted using a freshly prepared Oil Red O working solution (a 3:2 mixture of saturated Oil Red O-isopropanol solution and distilled water), followed by incubation for 8–10 min. Subsequently, the slides were briefly differentiated in 60% isopropanol, rinsed with distilled water, and counterstained with hematoxylin. Finally, the slides were mounted with glycerol gelatin for microscopic examination.

**Table 1:** RM2Target-based prediction of immune-related genes positively correlated METTL16 (Spearman r > 0.4) in Homo sapiens

Expression								
Target gene	Source	Log2FC	*P*-value	Adj.*P*-value	Perturbation direction	Perturbation effect	Method	GSE ID
PVR	NOMO-1	0.585	5.06E-03	2.04E-02	KO	up-regulated	MeRIP-seq	GSE189995
TNFSF13	NOMO-1	1.123	3.26E-02	8.81E-02	KO	up-regulated	MeRIP-seq	GSE189995
CXCL16	HEK293A	1.7669	2.93E-16	3.71E-14	KD	up-regulated	MeRIP-seq	GSE90914
CXCL16	HEK293T	0.7097	4.90E-02	1.59E-01	KO	up-regulated	MeRIP-seq	GSE182607
CXCL16	PANC-1	-1.229	2.83E-15	4.17E-14	KD	down-regulated	RNA-seq	GSE226518
HLA-DOA	HEK293A	1.5901	2.43E-02	8.33E-02	KD	up-regulated	MeRIP-seq	GSE90914
TAPBP	HEK293A	0.8258	8.61E-10	2.94E-08	KD	up-regulated	MeRIP-seq	GSE90914
TAPBP	Huh-7	0.7103	1.82E-27	3.09E-26	KO	up-regulated	RNA-seq	GSE224008
HLA-DRB1	HEK293T	1.0666	5.33E-05	6.88E-04	KO	up-regulated	MeRIP-seq	GSE182607
HLA-DRB1	NOMO-1	1.0335	1.15E-05	7.99E-05	KO	up-regulated	RNA-seq	GSE190044
HLA-DRB1	NOMO-1	1.0515	4.95E-04	3.10E-03	KO	up-regulated	MeRIP-seq	GSE189995
HLA-DRB1	NOMO-1	0.843	1.24E-03	4.32E-03	KO	up-regulated	MeRIP-seq	GSE189995
**Alternative splicing**								
**Target gene**	**Source**	**IncLevel difference**	**P-value**	**FDR**	**Perturbation direction**	**Alternative splicing type**	**Method**	**GSE ID**
PVR	Huh-7	-0.137	2.41E-04	2.12E-03	KO	A5SS	RNA-seq	GSE224008
PVR	Huh-7	0.367	9.21E-05	1.92E-03	KO	SE	RNA-seq	GSE224008
PVR	HEK293T	0.269	1.32E-06	4.59E-05	KO	SE	MeRIP-seq	GSE182607
PVR	NOMO-1	-0.396	2.43E-06	7.71E-05	KO	A5SS	MeRIP-seq	GSE189995
PVR	NOMO-1	-0.311	6.84E-06	1.73E-04	KO	A5SS	MeRIP-seq	GSE189995
TAPBP	Huh-7	-0.851	2.08E-08	1.56E-06	KO	SE	RNA-seq	GSE224008
NFKB1	AGS	0.16	1.14E-04	9.49E-03	KD	SE	MeRIP-seq	GSE224890
**Translation efficiency**								
**Target gene**	**Source**	**TE**	**Log2FC (RPF)**	**Log2FC (Input)**	**Perturbation direction**	**Perturbation effect**	**Method**	**GSE ID**
PVR	HEK293T	-3.8374	-3.0331	0.8043	KO	down-regulated	Ribo-seq	GSE156796
HLA-E	HEK293T	-0.8798	-0.1587	0.721	KO	down-regulated	Ribo-seq	GSE156796
**Binding evidence**								
**Target gene**	**Source**	**Log2FC**	**FPKM (Input)**		**FPKM (IP)**		**Method**	**GSE ID**
HLA-DRB1	HEK293T	3.1444	0.19		1.68		RIP-seq	GSE156797

To assess the expression of METTL16 and CIDEA, immunohistochemical staining was performed on paraffin-embedded liver tissue sections using antibodies against METTL16 (Proteintech, USA) and CIDEA (CUSABIO, China).

### RNA extraction, reverse transcription, and qPCR

Total RNA was isolated from mouse livers using TRIzol reagent. cDNA was synthesized from 1 µg of total RNA with random primers or oligo(dT) primers and reverse transcriptase following the manufacturer’s instructions. qPCR was performed with gene-specific primers using SYBR Green or TaqMan reagents under standard cycling conditions. The primer sequences used in this study were as follows: β-actin-F: 5’-GTGACGTTGACATCCGTAAAGA-3’, β-actin-R: 5’-GCCGGACTCATCGTACTCC-3’; TNFSF13-F: 5’-GCTGTCGCACTACTGATCCA-3’, TNFSF13-R: 5’-CAGGCTTCCAGGACATCAGG-3’; CXCL12-F: 5’-TGACGGTAAACCAGTCAGCC-3’, CXCL12-R: 5’-GTTCTTCAGCCGTGCAACAA-3’; CXCL16-F: 5’-GGGCTTTGGACCCTTGTCTCT-3’, CXCL16-R: 5’-AGAACAACTTCCAGCGACACT-3’.

### Western blot analysis

For protein extraction, HepG2 cells were lysed in RIPA buffer supplemented with protease inhibitors on ice. After sonication, the cell lysates were centrifuged at 12,000 rpm for 10 min at 4 °C. Protein concentrations were determined using the Bradford assay. Equal amounts of protein were mixed with loading buffer, denatured at 100 °C for 10 min, and then separated by SDS-PAGE. The proteins were subsequently transferred onto PVDF membranes. The membranes were blocked with 5% non-fat milk in TBST for 1 h at room temperature. Following blocking, the membranes were incubated with primary antibodies overnight at 4 °C, and then probed with HRP-conjugated secondary antibodies (Cell Signaling Technology, USA) for 1 h at 37 °C. After washing, protein bands were detected using enhanced chemiluminescence reagents and visualized via autoradiography. The primary antibodies and their dilutions used were as follows: METTL16 (Proteintech, USA) at 1:1000, CIDEA (CUSABIO, China) at 1:1000, and GAPDH (Cell Signaling Technology, USA) at 1:20000.

### Chromatin immunoprecipitation (ChIP) -qPCR

Mouse liver tissues were homogenized, followed by nuclei isolation and cross-linking with 1% formaldehyde. Chromatin was extracted and sonicated to generate DNA fragments of 200–1000 bp, then incubated with antibodies against EGR1 (Proteintech, USA) or MYC (Proteintech, USA). The resulting immune complexes were captured with Protein G magnetic beads, subjected to sequential washing steps, and eluted. Cross-links were reversed, and the recovered DNA was purified for subsequent qPCR analysis. qPCR was conducted with METTL16 fragment-specific primers and SYBR Green reagents under standard qPCR cycling conditions.

### Data sources and bioinformatic analyses

#### Hepatic gene expression profiles of MASLD patients and healthy controls

In the present study, the GSE135251 dataset from the publicly available GEO database, which includes hepatic gene expression profiles of 206 patients with MASLD and 10 healthy controls, was analyzed. Differentially expressed genes were identified using the DESeq2 package. Genes with an absolute log₂ fold change greater than 1 and an adjusted P-value less than 0.05 were considered statistically significant. Weighted Gene Co-expression Network Analysis (WGCNA) was performed using the WGCNA R package. Gene Ontology enrichment analysis of differentially expressed genes was performed using the ClusterProfiler package to identify significantly enriched biological processes. Additionally, Kyoto Encyclopedia of Genes and Genomes (KEGG) pathway analysis and Gene Set Enrichment Analysis (GSEA) were conducted via the ClusterProfiler package to explore potential pathways involved in MASLD progression. Spearman’s correlation analysis was applied to evaluate the correlation between METTL16 expression and genes related to immune responses and fibrosis.

#### Functional prediction of METTL16 on immune-related genes

Genes with Spearman correlation *r* > 0.4 with METTL16 in human datasets were selected as candidates. To systematically evaluate METTL16-mediated regulation at multiple layers the RM2Target database was utilized to predict potential regulatory effects of METTL16 on candidate genes, including the modulation of gene expression, alternative splicing, translation efficiency, and direct interactions.

#### MeRIP-seq analysis of METTL16-overexpressing HepG2 cells

To investigate the effects of METTL16 overexpression on m^6^A methylation, MeRIP-seq was performed on METTL16-overexpressing HepG2 cells and their corresponding control cells. The MeRIP-seq experiment and subsequent data analysis were conducted as previously described [25]. Additionally, KEGG pathway enrichment analysis was performed to predict the biological functions associated with differential m^6^A methylation sites.

#### Prediction of METTL16 regulatory factors

To predict potential transcription factors that regulate METTL16 expression, in silico analysis was carried out by integrating data from multiple databases, including ChIP-Atlas, GTRD, CHEA, ENCODE, PWMEnrich_JASPAR, FIMO_JASPAR, and hTFtarget.

#### Prediction of m^6^A methylation site on the CIDEA transcript

To identify potential m^6^A modification sites within the CIDEA transcript, SRAMP database was searched to pinpoint high-confidence m^6^A loci that might be functionally relevant to METTL16-mediated regulation.

#### Molecular docking of small-molecule compounds targeting human METTL16

Small-molecule compounds with potential targeting activity against human METTL16 were retrieved from the ChEMBL database. The 3D crystal structure of human METTL16 (PDB ID: 6M1U) and the selected compounds were preprocessed for molecular docking analysis. Blind docking was performed using the CB-Dock web server, which combines cavity detection with AutoDock Vina scoring. Docking simulations was run with default parameters, and binding affinity was quantified using the lowest Vina score (kcal/mol). Top-ranked docking poses (sorted by Vina score) were visualized and analyzed to identify key amino acid residues and potential interaction sites.

### Statistical analyses

All numerical data are presented as mean ± SD, and statistical analyses were conducted using GraphPad Prism 8. For comparisons between two independent groups, an unpaired Student’s t-test was applied. For comparisons involving three or more groups, one-way ANOVA was used to assess statistical significance. Temporal changes in GTT and ITT were analyzed by two-way ANOVA. A *P* value of < 0.05 was considered statistically significant.

## Results

### METTL16 plays a critical role in MASLD development

**Fig. 1:**
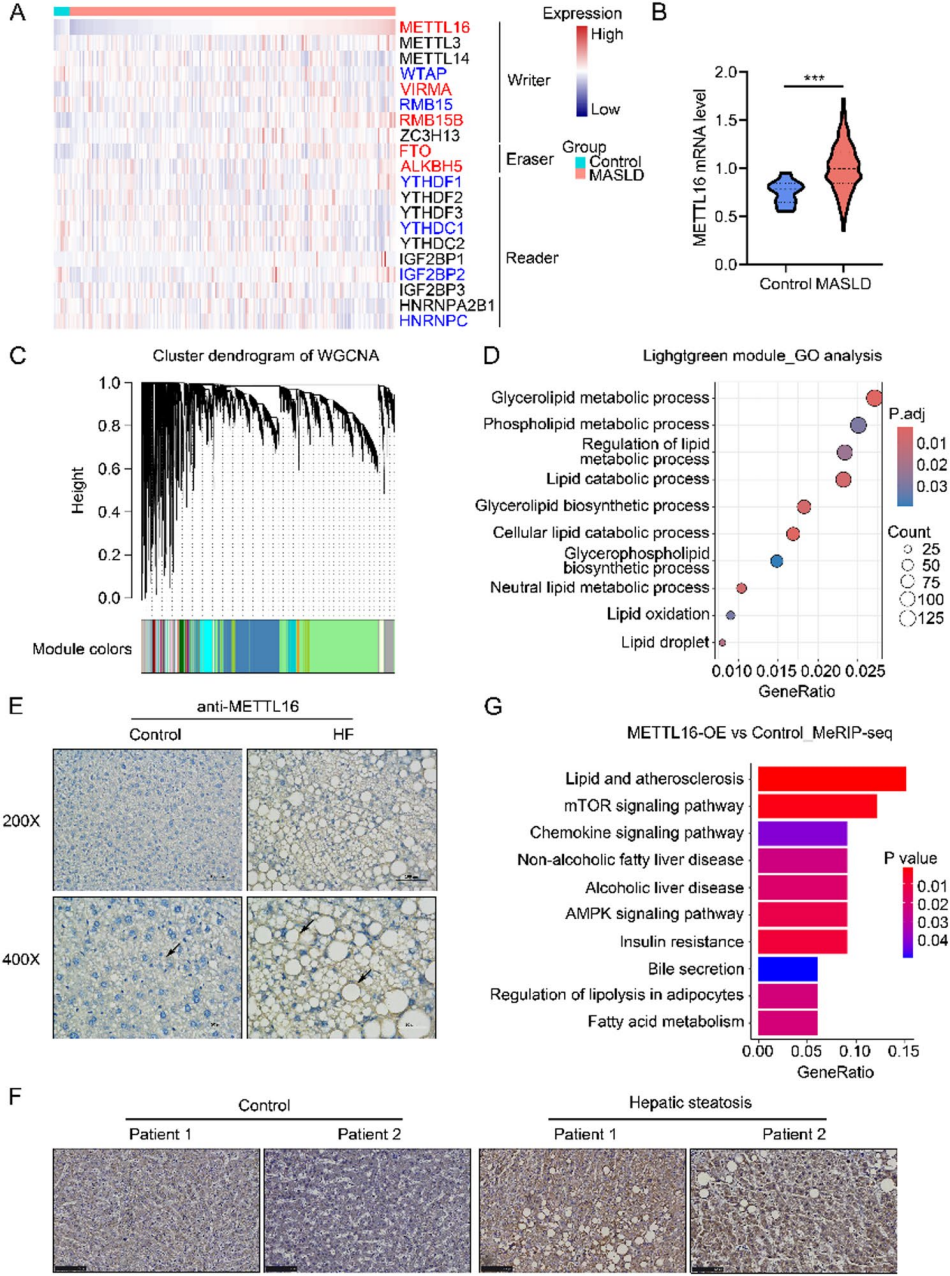
Expression profile and functional characterization of METTL16 in MASLD. (**A**) Heatmap displaying the normalized expression of m6A methylation writers, erasers, and readers in healthy individuals (*n*=10) and MASLD patients (*n*=206). Gene symbols highlighted in red indicate significant upregulation, while those in blue denote significant downregulation in MASLD patients relative to healthy controls (*P* < 0.05). (**B**) Comparison of METTL16 transcripts levels between MASLD patients and healthy individuals. (**C**) Gene co-expression modules identified by Weighted Gene Co-expression Network Analysis (WGCNA) in healthy individuals and MASLD patients. (**D**) Gene Ontology enrichment analysis of genes within the Lightgreen module. (**E-F**) Representative immunohistochemical staining of METTL16 in liver sections from HF diet-fed mice and chow-fed controls (**E**) or in human liver specimens with or without hepatic steatosis (**F**) (200×). (**G**) KEGG pathway enrichment analysis of differentially methylated genes identified by MeRIP-seq in METTL16-overexpressing HepG2 cells compared with empty vector controls. ***, *P* < 0.001

Based on the public GEO dataset (GSE135251), which includes a large cohort of participants (206 patients with MASLD and 10 healthy individuals), our analyses revealed a significant upregulation of methylation writers METTL16, VIRMA, and RMB15B, as well as methylation erasers FTO and ALKBH5 in MASLD patients (Fig. 1A, B, Figure S1A). In contrast, the expression levels of other methylation writers (WTAP, RMB15), and methylation readers (YTHDF1, YTHDC1, IGF2BP2, and HNRNPC) were significantly downregulated (Fig. 1A, Figure S1A). WGCNA was further performed, leading to the identification of 38 co-expression modules. Notably, METTL16 was clustered within the Lightgreen module, which was significantly enriched in lipid biosynthesis, catabolic, and metabolic pathways (Fig. 1C, D). Differential expression analysis identified 1637 upregulated genes and 554 downregulated genes between the high-METTL16 and low-METTL16 expression groups (Figure S1B). Pathway enrichment analysis of these differentially expressed genes further indicated that the non-alcoholic fatty liver disease pathway ranked among the top ten enriched KEGG pathways (Figure S1C). To validate these clinical observations, we established the MASLD animal model. Consistently, METTL16 expression was significantly upregulated in the livers of HF diet-fed mice (Fig. 1E) as well as in liver tissues from patients with hepatic steatosis (Fig. 1F). Complementary MeRIP-seq data demonstrated that METTL16 overexpression significantly altered the m^6^A methylation landscape across transcript regions in HepG2 cells (Figure S1D). Additionally, differentially methylated genes in the METTL16 overexpression group were enriched in the non-alcoholic fatty liver disease pathway compared to the control group (Fig. 1G). Collectively, these findings demonstrate that METTL16 plays a pivotal role in the regulation of lipid metabolism and strongly suggest its potential contribution to the pathogenesis of MASLD.

**Fig. 2:**
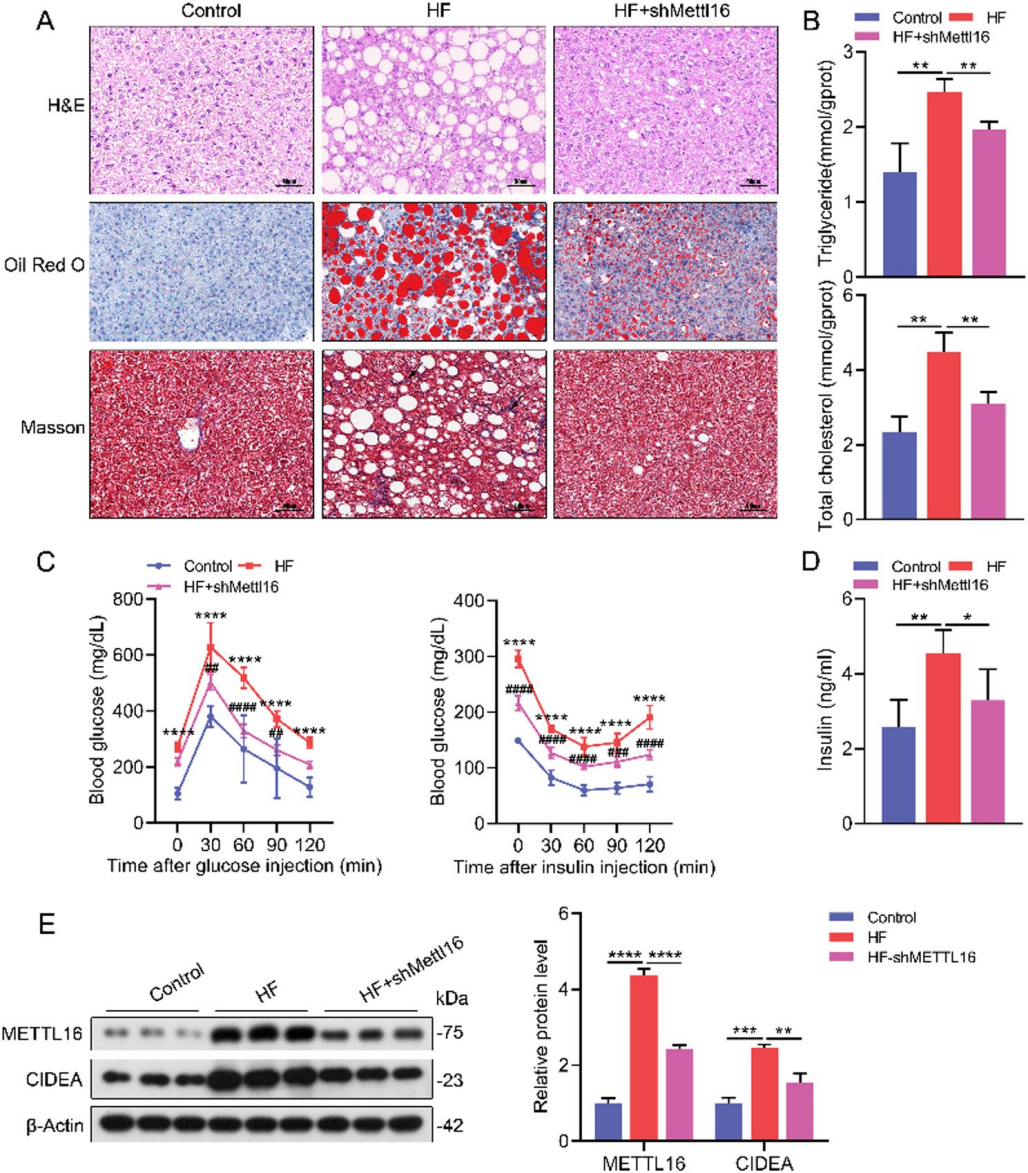
Mettl16 knockdown ameliorates lipid deposition, insulin resistance, and hepatic fibrosis in HF diet-fed mice. (**A**) Representative H&E, Oil Red O, and Masson staining of liver tissues (200×) from Mettl16 knockdown and wild-type mice fed a HF diet, and chow-fed controls, depicting hepatic morphology, steatosis, and fibrosis, respectively. (**B**-**D**) Serum cholesterol and triglyceride levels (**B**), glucose tolerance test (left panel) and insulin tolerance test (right panel) (**C**), and serum insulin levels (**D**) in HF diet-fed Mettl16 knockdown mice and other controls. (**E**) Western Blot analysis of METTL16 and CIDEA protein expression in livers from HF diet-fed Mettl16 knockdown mice and other controls. *, *P* < 0.05; **, *P* < 0.01; ***, *P* < 0.001; ****, *P* < 0.0001. For panel C: HF versus Control: ****, *P* < 0.0001; HF+shMettl16 versus HF: ^##^, *P* < 0.01; ^###^, *P* < 0.001; ^####^, *P* < 0.0001

**Fig. 3:**
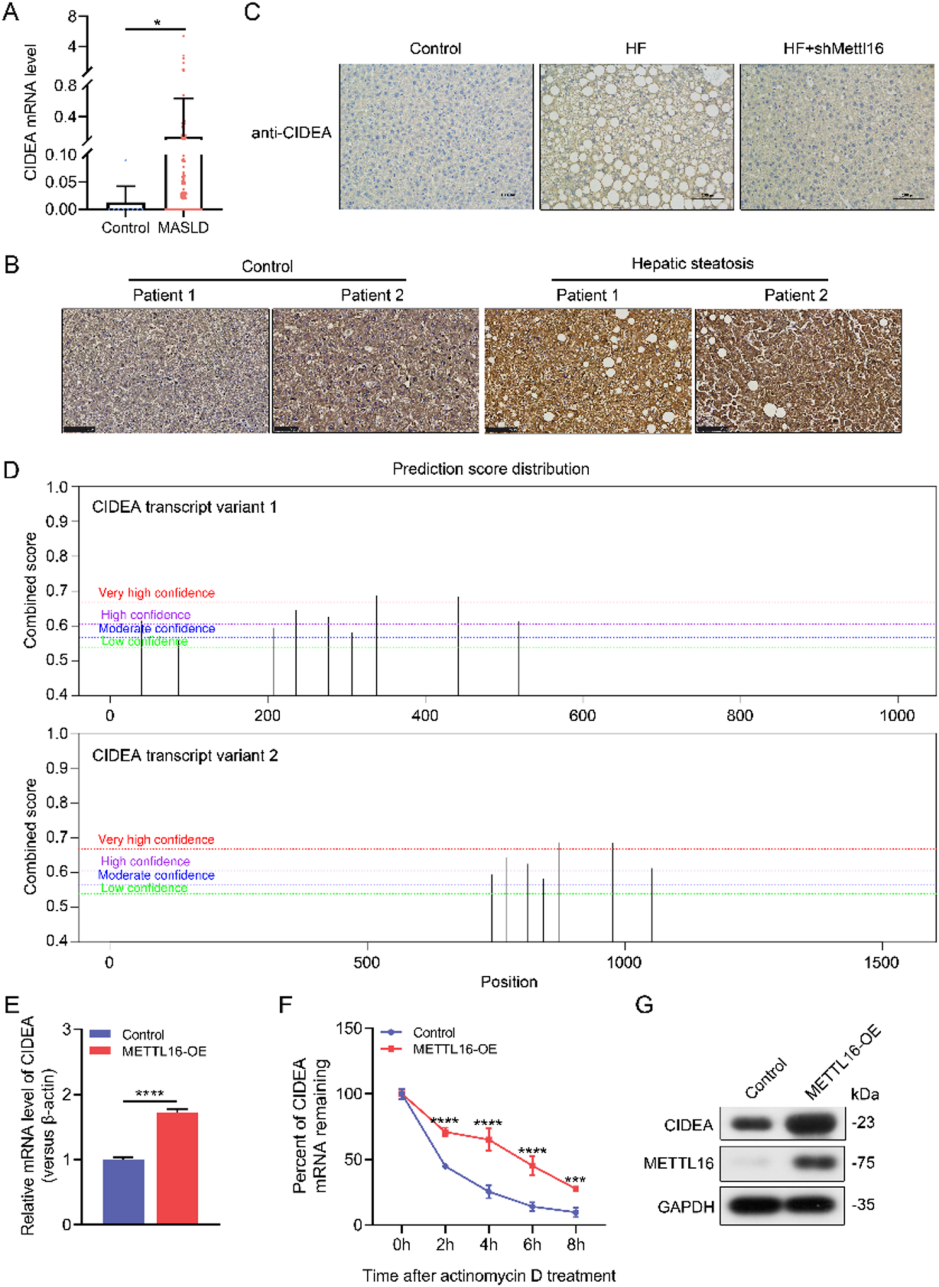
CIDEA is a potential downstream target of METTL16. (**A**) Levels of CIDEA transcripts in MASLD patients (*n* = 206) relative to healthy individuals (*n* = 10). (**B**) Representative immunohistochemical staining of CIDEA in human liver specimens with or without hepatic steatosis (200×). (**C**) Representative immunohistochemical staining of CIDEA in liver tissues from HF diet-fed Mettl16 knockdown mice and corresponding controls (200×). (**D**) Prediction of m^6^A methylation sites on the CIDEA transcripts using the SRAMP database. (**E**) Relative mRNA levels of CIDEA in METTL16-overexpressing HepG2 cells or controls. (**F**) Percentage of CIDEA mRNA remaining in METTL16-overexpressing HepG2 cells or controls following actinomycin D treatment (0, 2, 4, 6, and 8 h). (**G**) Western Blot analysis of CIDEA protein expression in METTL16 overexpressing HepG2 cells or controls. *, *P* < 0.05; ***, *P* < 0.001; ****, *P* < 0.0001

**Fig. 4:**
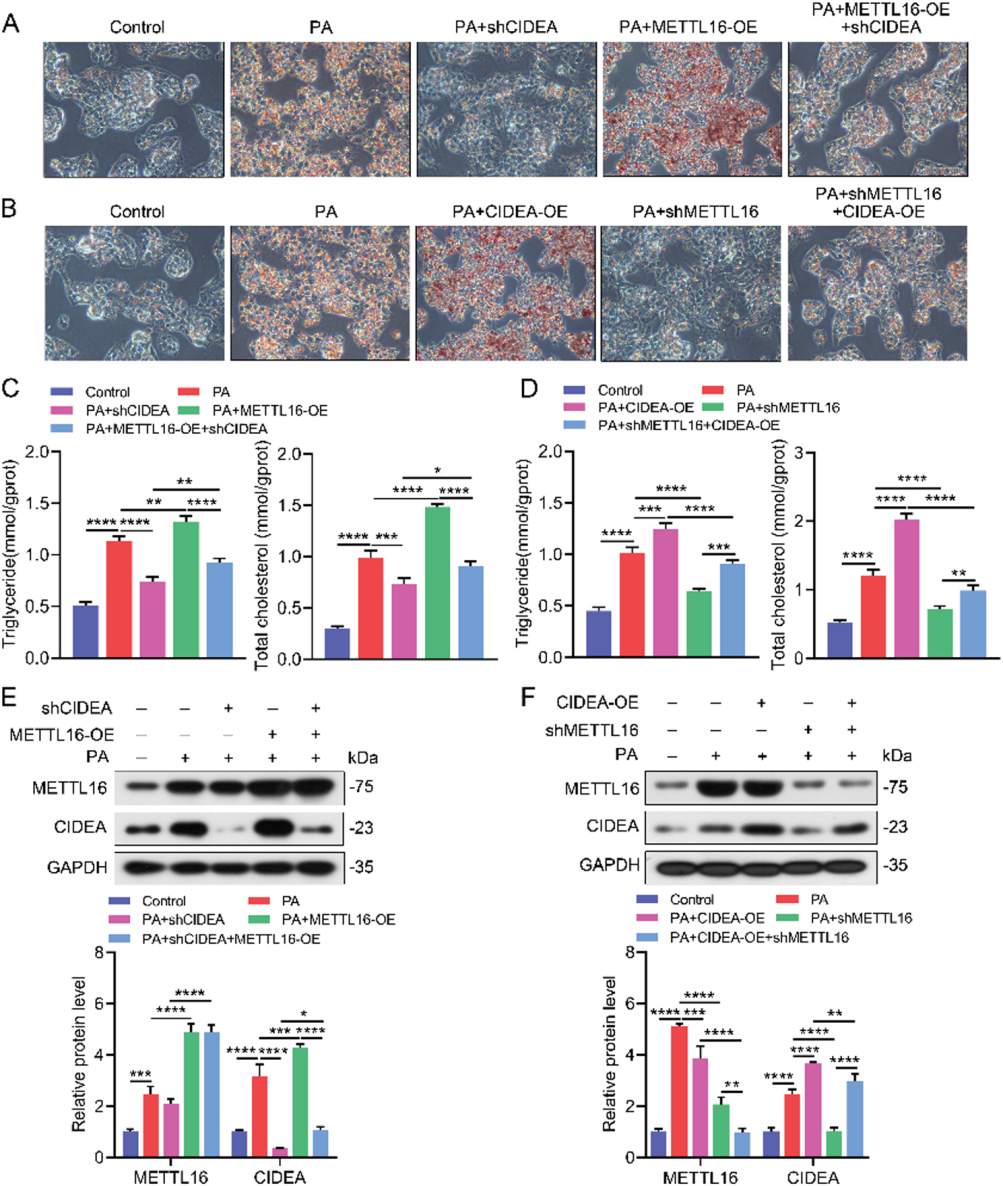
METTL16 regulates hepatic lipid metabolism through modulating CIDEA expression in a MASLD cell model. (**A**, **C**, **E**) Representative Oil Red O staining (**A**), intracellular cholesterol, triglyceride levels (**C**), and Western Blot analysis (**E**) of PA-induced HepG2 cells following METTL16 overexpression with or without CIDEA knockdown. (**B**, **D**, **F**) Representative Oil Red O staining (**B**), intracellular cholesterol, triglyceride levels (**D**), and Western Blot analysis of METTL16 and CIDEA (**F**) in PA-induced HepG2 cells following METTL16 knockdown with or without CIDEA overexpression. *, *P* < 0.05; **, *P* < 0.01; ***, *P* < 0.001; ****, *P* < 0.0001

**Fig. 5:**
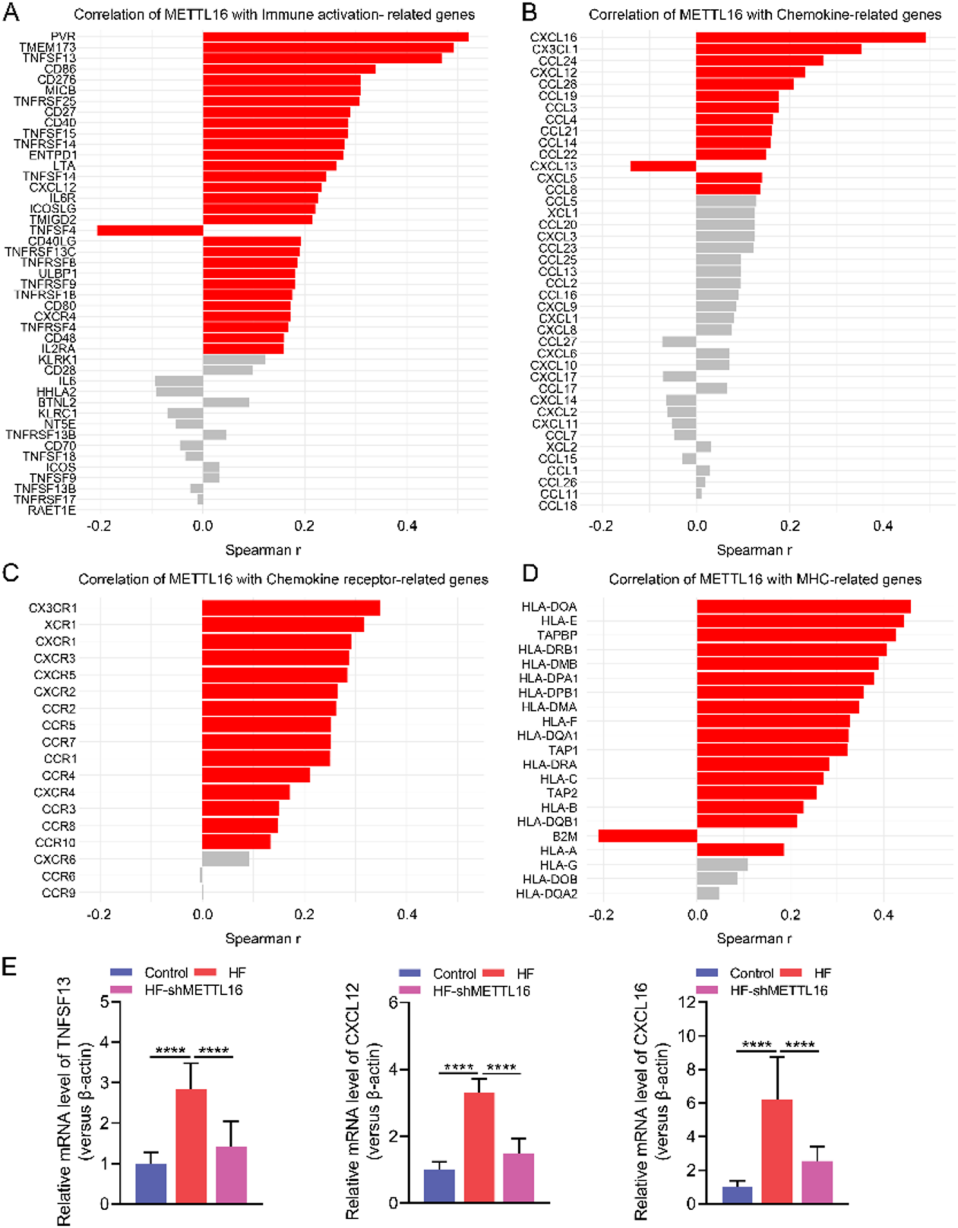
METTL16 exhibits a strong positive correlation with genes involved in immune response. Correlation analysis between METTL16 and genes associated with immune activation (**A**), chemokines (**B**), chemokine receptors (**C**), and major histocompatibility complex (MHC) molecules (**D**). Red bar indicates FDR < 0.05 of the correlation analysis. (**E**) Relative mRNA levels of TNFSF13, CXCL12 and CXCL16 in HF diet-fed Mettl16 knockdown mice and other controls. ****, *P* < 0.0001

**Fig. 6:**
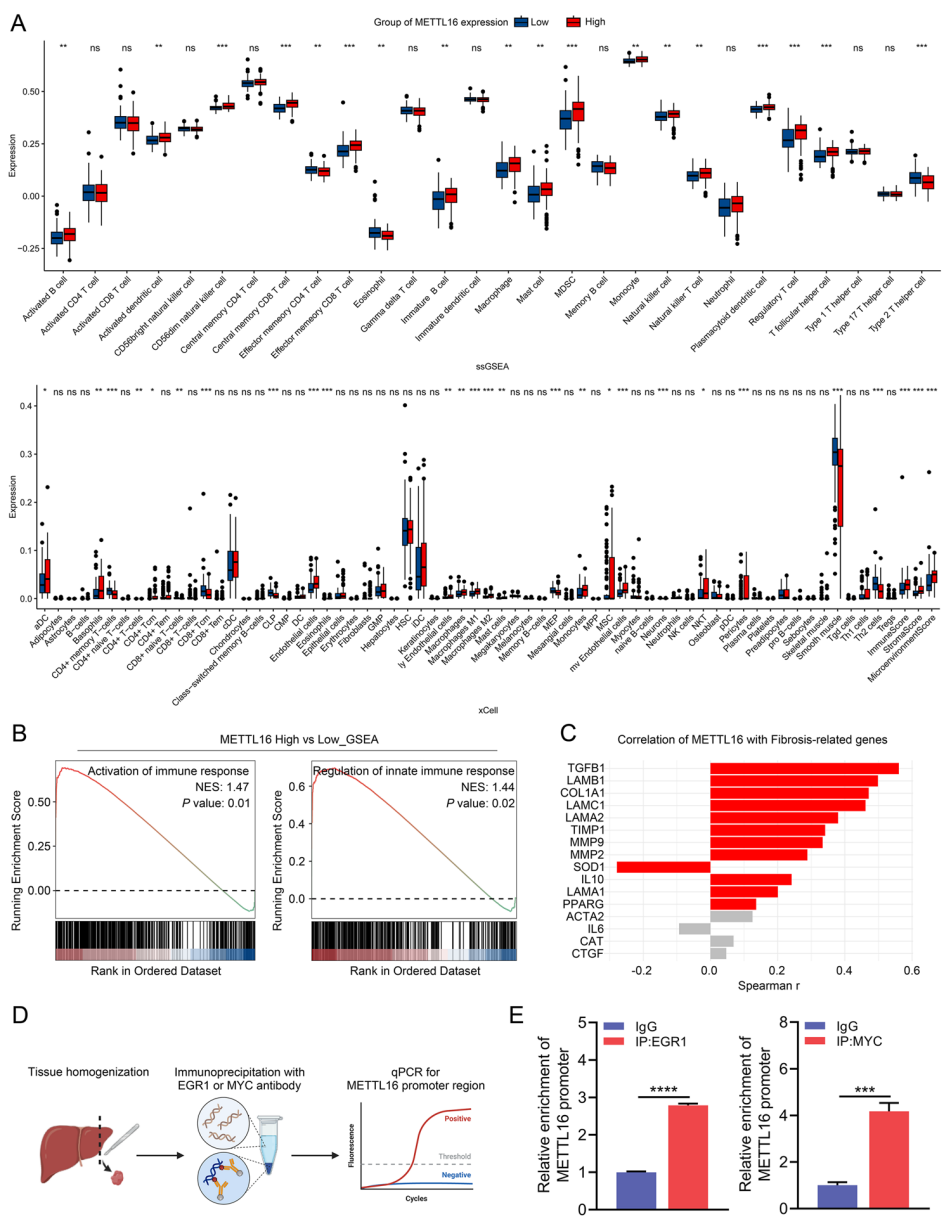
METTL16 facilitates MASLD progression through modulation of immune response. (**A**) ssGSEA and xCell analyses showing the relative abundance of immune cells in the high- versus low- METTL16 expression groups. (**B**) GSEA comparing the high- to the low- METTL16 expression group. (**C**) Correlation analysis between METTL16 expression and fibrosis-related genes. (**D**) Schematic diagram of ChIP-qPCR workflow. (**E**) Relative enrichment of METTL16 promoter region at EGR1- or MYC-bound chromatin. *, *P* < 0.05; **, *P* < 0.01; ***, *P* < 0.001; ****, *P* < 0.0001; ns, non-significant

**Fig. 7:**
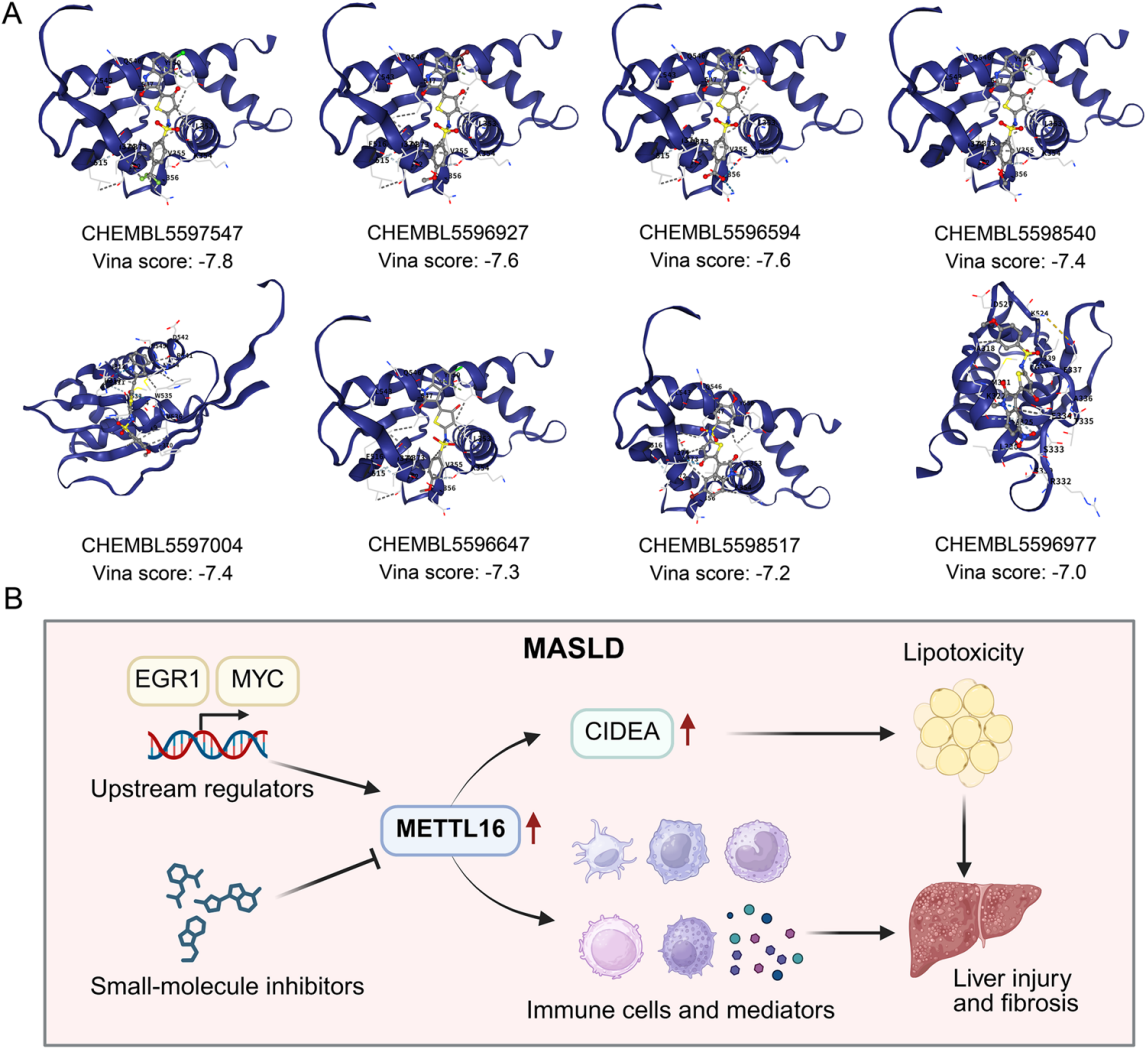
Identification and molecular docking analysis of METTL16 inhibitors. (**A**) Molecular docking analysis and Vina scores of METTL16 and 8 small-molecule inhibitors searched from ChEMBL database. (**B**) A proposed model of METTL16-mediated effects on MASLD pathogenesis. In MASLD, METTL16 is markedly upregulated in hepatocytes, promoting CIDEA expression and thereby enhancing lipid accumulation and lipotoxicity. Additionally, METTL16 modulates the activation of immune cells and the production of immune mediators within the liver, exacerbating immune-inflammatory responses. These processes collectively drive hepatic inflammation and fibrosis. EGR1 and MYC act as potential transcriptional regulators of METTL16. Small-molecule inhibitors targeting METTL16 or its associated signaling pathways could represent promising therapeutic strategies for MASLD

### Mettl16 knockdown ameliorates lipid deposition, insulin resistance, and liver fibrosis in HF diet-fed mice

To further dissect the functional the role of METTL16 in MASLD pathogenesis, we established a Mettl16 knockdown mouse model and subjected these mice to a HF diet. Food intake did not differ between HF diet-fed Mettl16 knockdown mice and control mice (Figure S2A). However, HF diet-fed mice displayed a significant increase in body weight, liver weight, and liver-to-body weight ratio, all of which were markedly attenuated by METTL16 knockdown (Figure S2B). After HF diet feeding, lipid and collagen deposition in liver tissues were significantly aggravated, as evidenced by Oil Red O staining and Masson staining (Fig. 2A). In stark contrast, Mettl16 knockdown mice exhibited markedly smaller lipid droplets and a significant reduction in collagen fiber deposition in liver tissues (Fig. 2A), indicating alleviated hepatic steatosis and fibrosis. Consistently, serum biochemical assays demonstrated that METTL16 knockdown significantly reduced circulating levels of TC and TG (Fig. 2B). GTT and ITT results showed that Mettl16 knockdown mice exhibited significant improvement in glucose homeostasis and insulin resistance following HF diet challenge (Fig. 2C, Figure S2C, D). Furthermore, the serum insulin levels were markedly reduced in Mettl16 knockdown mice, confirming the improvement in systemic insulin resistance (Fig. 2D). Notably, the expression of METTL16 and the lipid metabolism-related gene CIDEA was elevated in HF diet-fed mice (Fig. 2E). Following METTL16 knockdown, CIDEA expression was markedly downregulated (Fig. 2E).

### CIDEA is a potential downstream molecule of METTL16

CIDEA is a well-characterized lipogenic gene, that belongs to the CIDE family and modulates lipid droplet formation and lipid metabolism homeostasis. Based on the publicly available GEO dataset (GSE135251), we observed that CIDEA expression was elevated in MASLD patients compared to healthy individuals (Fig. 3A). Consistent with this observation, immunohistochemical staining demonstrated a marked upregulation of CIDEA expression in liver tissues from patients with hepatic steatosis (Fig. 3B). Notably, CIDEA protein levels were also increased in the liver tissues of HF diet-fed mouse models, and after METTL16 knockdown, CIDEA expression was reduced (Fig. 3C). Consistently, CIDEA protein expression was significantly increased in METTL16 overexpressing HepG2 cells compared to controls (Fig. 3G, Figure S3). Moreover, using the SRAMP database, multiple m^6^A methylation sites were identified on the CIDEA transcripts (Table S1, Fig. 3D). These results suggest that METTL16 may play a role in MASLD by regulating CIDEA expression. To elucidate the molecular mechanism underlying METTL16-mediated regulation of CIDEA, we assessed CIDEA expression in METTL16-overexpressing HepG2 cells. METTL16 overexpression led to a significant increase in CIDEA mRNA levels (Fig. 3E). Treatment with actinomycin D further demonstrated that METTL16 attenuated the degradation of CIDEA mRNA, indicating enhanced stability of the CIDEA transcript (Fig. 3F), which was accompanied by an increase in CIDEA protein expression (Fig. 3G).

### METTL16 regulates hepatic lipid metabolism by modulating CIDEA expression in MASLD

To further investigate the regulatory mechanisms of METTL16 on CIDEA, we established a cell model of MASLD by inducing HepG2 cells with PA, which was a well-validated in vitro approach to mimic hepatic steatosis by promoting intracellular lipid accumulation. Oil Red O staining showed that overexpression of CIDEA increased hepatic lipid deposition in PA-induced HepG2 cells, whereas knockdown of CIDEA exerted the opposite effect (Fig. 4A, B). To confirm whether METTL16 regulates lipid metabolism through CIDEA, we performed rescue experiments. Overexpression of METTL16 resulted in increased lipid deposition. However, when CIDEA was knocked down in METTL16 overexpressing PA-induced HepG2 cells, lipid deposition was significantly reduced (Fig. 4A). Conversely, knockdown of METTL16 led to decreased lipid deposition, while overexpression of CIDEA in METTL16-knockdown HepG2 cells increased lipid deposition (Fig. 4B). Intracellular cholesterol and triglyceride levels were agreed with above findings (Fig. 4C, D). Western blot analysis also revealed that both METTL16 and CIDEA expression were significantly elevated upon PA treatment (Fig. 4E, F). Overexpression of METTL16 led to a substantial increase in CIDEA expression, while knockdown of METTL16 resulted in reduced CIDEA expression, suggesting that METTL16 acts as an upstream regulator of CIDEA (Fig. 4E, F). These results indicate that METTL16 regulates lipid metabolism in MASLD by modulating CIDEA expression.

### METTL16 facilitates MASLD progression through the modulation of immune response

Given the significant alleviation in liver fibrosis upon METTL16 knockdown (Fig. 2A), we performed correlation analyses between METTL16 and immune-related markers using public GEO dataset (GSE135251). We found that METTL16 was significantly positively correlated with genes associated with immune activation (Fig. 5A), chemokines (Fig. 5B), chemokine receptors (Fig. 5C), and major histocompatibility complex (Fig. 5D), suggesting that immune responses might contribute to METTL16-mediated MASLD progression. To further validate these observations, we performed qPCR analysis on liver tissues from HF diet-fed mice to assess the transcriptional levels of representative immune-associated genes, including TNFSF13, CXCL12, and CXCL16. In mice fed with a HF diet, the transcriptional levels of these genes were markedly upregulated, whereas METTL16 knockdown significantly blunted their expression (Fig. 5E). Furthermore, METTL16 expression showed a significant positive correlation with NFKB1, which encodes the p50 subunit of NF-κB, a key regulator of immune and inflammatory responses [26] (Figure S4A). Furthermore, RM2Target predictions for immune-related genes with Spearman correlation *r* > 0.4 with METTL16 indicated that in human cell lines, METTL16 may regulate the gene expression of PVR, TNFSF13, CXCL16, HLA-DOA, TAPBP, and HLA-DRB1; modulate the alternative splicing of PVR, TAPBP and NFKB1; affect the translation efficiency of PVR and HLA-E; and directly interact with HLA-DRB1 (Table 1).

**Table 2:** Characteristics of small-molecule inhibitors of METTL16 identified from the ChEMBLdatabase

ChEMBL ID	Molecular Weight	Targets	Bioactivities	AIogP	Polar Surface Area	HBA	HBD	#RO5 Violations	#RotatableBonds	PassesRo3	QED Weighted
CHEMBL5598540	428.49	1	1	2.8	101.57	6	2	0	4	N	0.73
CHEMBL5598517	507.39	1	1	3.28	92.78	6	1	1	4	N	0.64
CHEMBL5597547	486.88	1	1	4.16	92.34	5	2	0	3	N	0.64
CHEMBL5597004	412.49	1	1	3.65	88.26	5	2	0	5	N	0.62
CHEMBL5596977	473.49	1	1	2.43	135.92	8	1	0	5	N	0.4
CHEMBL5596927	493.36	1	1	3.26	101.57	6	2	0	4	N	0.63
CHEMBL5596647	448.91	1	1	3.15	101.57	6	2	0	4	N	0.7
CHEMBL5596594	507.34	1	1	2.95	129.64	6	3	1	4	N	0.54
CHEMBL5414547	401.42	12	17	0.19	145.78	10	5	0	7	N	0.35
CHEMBL5411006	492.58	12	17	1.28	155.09	11	4	1	7	N	0.42
CHEMBL5406914	478.55	12	17	0.89	155.09	11	4	1	6	N	0.47

ssGSEA and xCell analyses revealed that the abundance of innate immune cells, including activated dendritic cells (aDC), macrophages, monocytes, natural killer T (NKT) cells, and mast cells, along with ImmuneScore, StromalScore, and MicroenvironmentScore were significantly elevated in the high METTL16 expression group, whereas Th2 cells were decreased (Fig. 6A). When samples were stratified into high and low METTL16 expression groups, Gene Ontology enrichment analysis of differentially expressed genes showed significant enrichment in various immune and lipid metabolism pathways (Figure S4B). Furthermore, GSEA indicated that the “Activation of immune response” and “Regulation of innate immune response” pathways were significantly activated in the high METTL16 expression group (Fig. 6B). Additionally, correlation analysis demonstrated a significant positive correlation between METTL16 expression and pro-fibrotic genes (TGFB1, COL1A1, LAMB1, LAMC1), with correlation coefficients greater than 0.4 (Fig. 6C).

To further identify potential transcription factors regulating METTL16, in silico analyses were performed using multiple databases, including ChIP-Atlas, GTRD, CHEA, ENCODE, PWMEnrich_JASPAR, FIMO_JASPAR, and hTFtarget. Notably, EGR1 and MYC were consistently predicted as a major transcriptional regulator of METTL16 (Figure S4C). To investigate the potential binding of EGR1 and MYC to the METTL16 promoter, we performed ChIP-qPCR assays using mouse livers (Fig. 6D). As presented in Fig. 6E, both EGR1 and MYC were significantly enriched at the METTL16 promoter compared with the IgG control, supporting direct association of EGR1 and MYC with the METTL16 promoter in vivo.

### Identification of selective small-molecule inhibitors targeting METTL16 as potential therapeutics for MASLD

Given that the above results confirmed the role of METTL16 in promoting MASLD progression, we sought to identify potential targeted inhibitors. Screening the ChEMBL database identified 11 small-molecule inhibitors of human METTL16, 8 of which were selective, indicating high specificity and utility for targeted therapeutic development (Table 2).

Molecular docking analysis showed that the Vina scores of these eight compounds ranged from − 7.0 to -9.0 kcal/mol, suggesting favorable binding affinities with METTL16 and promising inhibitory effects on its activity (Fig. 7A). Among these, compound CHEMBL5597547 exhibited the lowest Vina score, implying the strongest predicted binding affinity and potential as a therapeutic candidate for MASLD.

## Discussion

METTL proteins are characterized by a distinctive domain that facilitates methyl transfer reactions using the methyl donor, S-adenosylmethionine (SAM). As a recently identified m⁶A RNA methyltransferase, METTL16 acts independently of the METTL3-METTL14 core complex and harbors an N-terminal methyltransferase domain (MTD) along with a C-terminal region containing two vertebrate-conserved regions (VCRs) [27]. Previous studies have shown the involvement of METTL16 in the development and progression of hepatocellular carcinoma [28–30] and cholangiocarcinoma [31]. However, its role in other liver diseases, particularly in MASLD, remains poorly understood.

In the current study, we first performed a comprehensive analysis of the expression profiles of methylation-related genes in 206 MASLD patients and 10 healthy controls, which revealed that METTL16 is significantly upregulated in MASLD patients. Additionally, we observed that in the “writer” category, VIRMA and RBM15 were upregulated, while WTAP and RBM15B were downregulated. In the “eraser” category, FTO and ALKBH5 were upregulated, and in the “reader” category, YTHDF1, YTHDC1, IGF2BP2, and HNRNPC were downregulated. These findings showed significant overlap with a previous study based on a dataset comprising 24 healthy controls and 30 MASLD patients [7]. Notably, no significant changes in the transcriptional levels of METTL3/14 were observed in this study. This discrepancy may be attributed to differences in sample size and the sequencing method. The previous study used microarray sequencing [7], whereas the dataset used in our study employed high-throughput RNA sequencing. Whether the expression levels of methylation-related genes are influenced by specific clinical or metabolic features in MASLD patients, such as increased body weight, insulin resistance, hepatic steatosis severity and lobular inflammation, remains to be further investigated.

Subsequent integrative analyses, including WGCNA, MeRIP-seq, and immunohistochemical staining conducted on both mouse and human liver specimens, indicated that METTL16 is closely implicated in lipid metabolism. Our previous study demonstrated upregulated METTL16 expression in both in vivo and in vitro MASLD models [25], consistent with findings in the livers of type 2 diabetes mellitus rats induced by HF diet feeding and intraperitoneal streptozotocin injection [32]. In the present study, we showed that METTL16 knockdown in MASLD mouse models alleviated hepatic steatosis, insulin resistance, and fibrosis, underscoring its role in disease progression.

CIDEA has been shown to correlate closely with the severity of hepatic steatosis by regulating AMP-activated protein kinase stability [33]. In addition, our previous study revealed marked elevation of both m^6^A methylation and expression levels of CIDEA in the livers of HF diet-fed mice [25]. Herein, CIDEA was identified as a key mediator of METTL16-driven hepatic steatosis in MASLD, as confirmed by METTL16 overexpression and CIDEA knockdown experiments. Analysis with the SRAMP database predicted multiple putative methylation sites on the CIDEA transcript. Furthermore, METTL16 enhanced the stability of CIDEA mRNA, indicating that it regulates CIDEA expression, at least in part, at the post-transcriptional level. It has been reported that METTL16 regulates gene expression through both m^6^A-dependent and m^6^A-independent mechanisms [34]. In the nucleus, it functions as an m^6^A “writer”, introducing m^6^A modifications to hundreds of specific mRNA targets [34]. In the cytoplasm, METTL16 can enhance translation independently of m^6^A, interacting with eukaryotic initiation factors 3 A and 3B as well as ribosomal RNA via its methyltransferase domain, thereby facilitating assembly of the translation-initiation complex and promoting translation of thousands of mRNA transcripts [27, 34]. Whether METTL16 regulates CIDEA through an m^6^A- independent mechanism requires further investigation.

Intriguingly, hepatic METTL16 expression was positively correlated with the levels of immune mediators and pro-fibrotic genes. Notably, METTL16 preferentially targets U6 small nuclear RNA (snRNA) and SAM synthetase precursor mRNA, thereby distinguishing it from other m^6^A writers such as METTL3 and METTL14. The conserved VCRs of METTL16 catalyzes the m^6^A modification at position 43 within U6 snRNA. As a core component of the spliceosome, U6 snRNA is indispensable for catalyzing intron excision from pre-mRNA. Furthermore, METTL16 shapes the m^6^A epitranscriptome by sustaining SAM homeostasis through its N-terminal MTD [27]. Analysis based on the RM2Target database predicted that METTL16 regulated the protein expression, alternative splicing, and translation efficiency of multiple immune-related genes, and even directly interacted with HLA-DRB1. These findings suggest that METTL16 may participate in the immunopathological processes of MASLD at both transcriptional and post-transcriptional levels. Notably, several innate immune cell populations, including aDC, macrophages, monocytes, NKT cells, and mast cells, were more abundant in samples with high METTL16 expression. Furthermore, METTL16 expression was positively associated with the activation of various immune-related signaling pathways. These observations are consistent with previous reports that expansion and activation of NKT cells and dendritic cells promote hepatic inflammation and liver injury during MASH, while recruited monocytes can differentiate into pro-inflammatory macrophages or monocyte-derived Kupffer cells, amplifying hepatic immune responses [35]. Importantly, mast cells have been shown to exacerbate biliary and hepatic injury and may facilitate the development of microvesicular steatosis, thereby potentially contributing to simple steatosis progression toward MASH [36]. The mechanisms underlying how METTL16 regulates the function of these innate immune cells in the progression of MASLD warrant further investigation. 

To explore the therapeutics targeting METTL16 for MASLD, upstream transcription factors were predicted through multiple databases, identifying EGR1 and MYC as potential regulators of METTL16. Additionally, eleven small-molecule compounds were predicted to bind with METTL16 and inhibit its activity, among which eight compounds have been reported to selectively target METTL16, suggesting high specificity and reduced off-target effects. Furthermore, a novel cinnamic acid derivative has been reported to induce degradation of METTL16 protein in the context of hepatocellular carcinoma [37]. Two pioneering multi-target inhibitors against various methyltransferase-like proteins have also been demonstrated to suppress the proliferation of liver cancer cells [38]. Taken together, these findings underscore METTL16 as a critical epigenetic regulator in MASLD and highlight its potential as a therapeutic target. However, the therapeutic potential and safety of these compounds for MASLD remain to be validated in cellular and animal models.

## Supplementary Information

Below is the link to the electronic supplementary material.

Supplementary Material 1

## Data Availability

The RNA‐seq datasets were downloaded from http://www.ncbi.nlm.nih.gov// with the accession number GSE135251. Data related to this paper are available on reasonable request.

## Abbreviations

MASLD: Metabolic dysfunction-associated steatotic liver disease
MASH: Metabolic dysfunction-associated steatohepatitis
m^6^A: N^6^-methyladenosine
METTL3: Methyltransferase-like 3
FTO: Fat mass and obesity-associated protein
WTAP: Wilms tumor 1-associated protein
RBM15: RNA-binding motif protein 15
YTHDC1: YT521-B homology domain-containing 1
IGF2BP1: Insulin-like growth factor 2 mRNA binding protein 1
HNRNPC: Heterogeneous nuclear ribonucleoprotein C
CIDEA: Cell death-inducing DFF45 like effector family members A
PA: Palmitic acid
HF: High-fat
GTT: Glucose tolerance test
ITT: Insulin tolerance test
TG: Triglyceride
TCHO: Total cholesterol
H&E: Hematoxylin & eosin
WGCNA: Weighted Gene Co-expression Network Analysis
KEGG: Kyoto Encyclopedia of Genes and Genomes
GSEA: Gene Set Enrichment Analysis
NKT: Natural killer T
aDC: Activated dendritic cells
SAM: S-adenosylmethionine
VCRs: Vertebrate-conserved regions
MTD: N-terminal methyltransferase domain
snRNA: Small nuclear RNA
