# Supplementary material for: METTL16 promotes MASLD progression through the regulation of lipid synthesis and immune response
Source: Biol Direct. 2026 Apr 9;21:40. doi: 10.1186/s13062-026-00760-0 (PMC13063659; doi:10.1186/s13062-026-00760-0)
Supplement: Supplementary file 1 — Supplementary Material 1 [file 13062_2026_760_MOESM1_ESM.docx]

**Supplementary materials**

**METTL16 promotes MASLD progression through the regulation of lipid synthesis and immune response**

| No. | Position | Sequence context | Score(binary) | Score(knn) | Score(spectrum) | Score(combined) | Decision |
| --- | --- | --- | --- | --- | --- | --- | --- |
| NM_001279.4 CIDEA, transcript variant 1, mRNA | | | | | | | |
| 1 | 40 | CGCGCCAUGGAGGCCGCCCGGGACUAUGCAGGAGCCCUCAUCAGG | 0.666 | 0.855 | 0.513 | 0.614 | High confidence |
| 2 | 87 | CCUGACAUUUAUGGGAUCACAGACUAAGCGAGUCCUGUUCACCCC | 0.573 | 0.709 | 0.519 | 0.558 | Low confidence |
| 3 | 207 | CCUGCAGGAGCUCAUCAGCAAGACUCUGGAUGCCCUCGUCAUCGC | 0.574 | 0.744 | 0.601 | 0.593 | Moderate confidence |
| 4 | 236 | AUGCCCUCGUCAUCGCUACCGGACUGGUCACUCUGGUGCUGGAGG | 0.667 | 0.763 | 0.599 | 0.644 | High confidence |
| 5 | 277 | GAGGAAGAUGGCACCGUGGUGGACACAGAAGAGUUCUUUCAGACC | 0.576 | 0.702 | 0.682 | 0.625 | High  confidence |
| 6 | 307 | GAGUUCUUUCAGACCUUGGGAGACAACACGCAUUUCAUGAUCUUG | 0.481 | 0.470 | 0.733 | 0.581 | Moderate  confidence |
| 7 | 338 | AUUUCAUGAUCUUGGAAAAAGGACAGAAGUGGAUGCCGGGCAGCC | 0.619 | 0.666 | 0.781 | 0.686 | Very high  confidence |
| 8 | 442 | UUGUACAGGCUGAACCCCAAGGACUUCAUCGGCUGCCUUAACGUG | 0.681 | 0.700 | 0.686 | 0.684 | Very high confidence |
| 9 | 518 | CCUACGACAUCCGGUGCACGGGACUCAAGGGCCUGCUGAGGAGUC | 0.616 | 0.468 | 0.625 | 0.612 | High confidence |
| NM_001318383.2 CIDEA, transcript variant 2, mRNA | | | | | | | |
| 1 | 742 | CCUGCAGGAGCUCAUCAGCAAGACUCUGGAUGCCCUCGUCAUCGC | 0.574 | 0.744 | 0.601 | 0.593 | Moderate  confidence |
| 2 | 771 | AUGCCCUCGUCAUCGCUACCGGACUGGUCACUCUGGUGCUGGAGG | 0.667 | 0.763 | 0.599 | 0.644 | High confidence |
| 3 | 812 | GAGGAAGAUGGCACCGUGGUGGACACAGAAGAGUUCUUUCAGACC | 0.576 | 0.702 | 0.682 | 0.625 | High confidence |
| 4 | 842 | GAGUUCUUUCAGACCUUGGGAGACAACACGCAUUUCAUGAUCUUG | 0.481 | 0.470 | 0.733 | 0.581 | Moderate  confidence |
| 5 | 873 | AUUUCAUGAUCUUGGAAAAAGGACAGAAGUGGAUGCCGGGCAGCC | 0.619 | 0.666 | 0.781 | 0.686 | Very high confidence |
| 6 | 977 | UUGUACAGGCUGAACCCCAAGGACUUCAUCGGCUGCCUUAACGUG | 0.681 | 0.700 | 0.686 | 0.684 | Very high confidence |
| 7 | 1053 | CCUACGACAUCCGGUGCACGGGACUCAAGGGCCUGCUGAGGAGUC | 0.616 | 0.468 | 0.625 | 0.612 | High confidence |

**T****able S1. SRAMP**-**based prediction of m^6^A sites in liver-derived CIDEA mRNA**


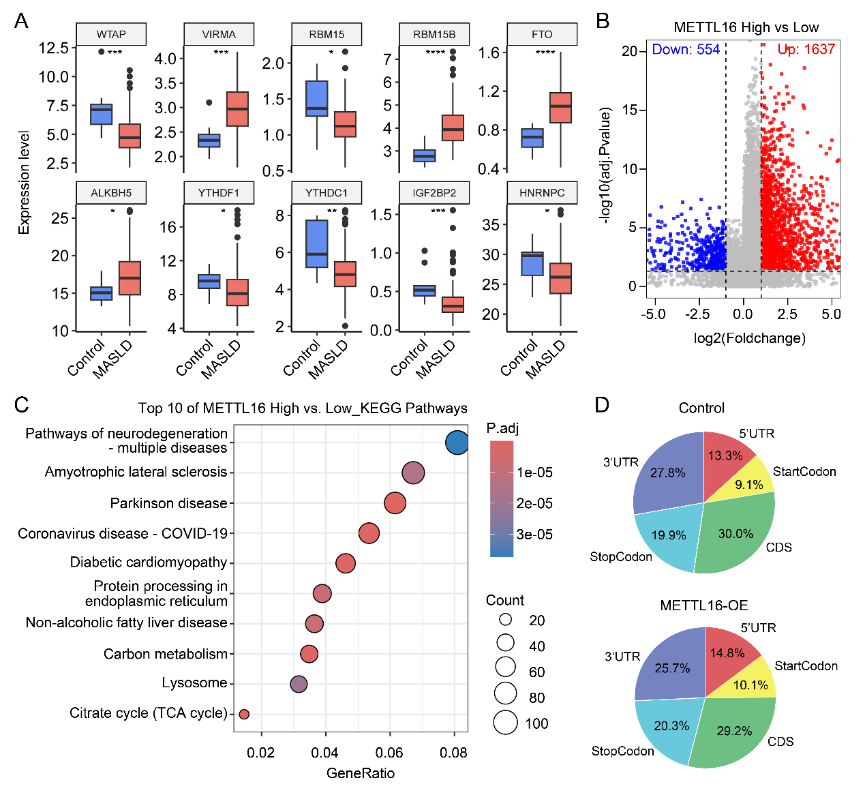
**Figure S1. Expression profile and functional analysis of METTL16 in MASLD.** (A) Expression of methylation writers, erasers, and readers in patients with MASLD compared to healthy individuals. (B) Volcano plot showing differentially expressed genes between groups with high and low METTL16 expression. (C) KEGG pathway enrichment analysis of differentially expressed genes stratified by METTL16 expression level (high vs. low). (D) MeRIP-seq analysis illustrating distribution of methylation peaks across transcript regions in Control and METTL16 overexpression groups. *, *P*<0.05; **, *P*<0.01; ***, *P*<0.001; ****, *P*<0.0001.


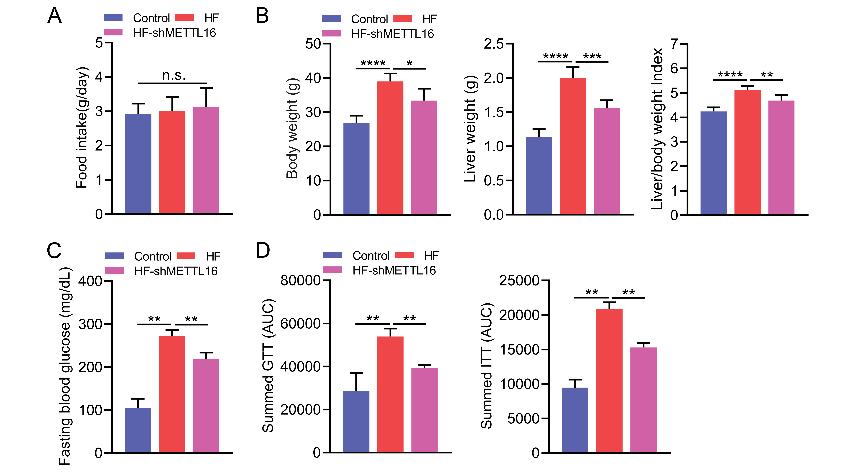
**Figure S2.** **Glucose metabolism and insulin sensitivity in mice fed a HF diet.** Parameters shown include food intake (A), body weight, liver weight, liver-to-body weight ratio (B), fasting blood glucose levels (C), as well as the area under the curve (AUC) for glucose tolerance test (GTT) and insulin tolerance test (ITT) (D) in mice subjected to 16-week high-fat feeding with Mettl16 knockdown and alongside corresponding control groups.


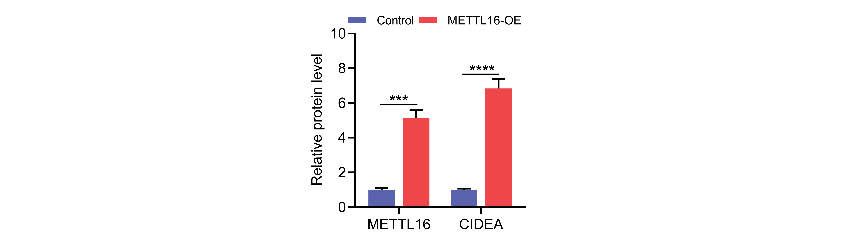
**Figure S3. CIDEA is a potential downstream molecule of METTL16.** Western Blot analysis of CIDEA protein expression in METTL16-overexpressing HepG2 cells.


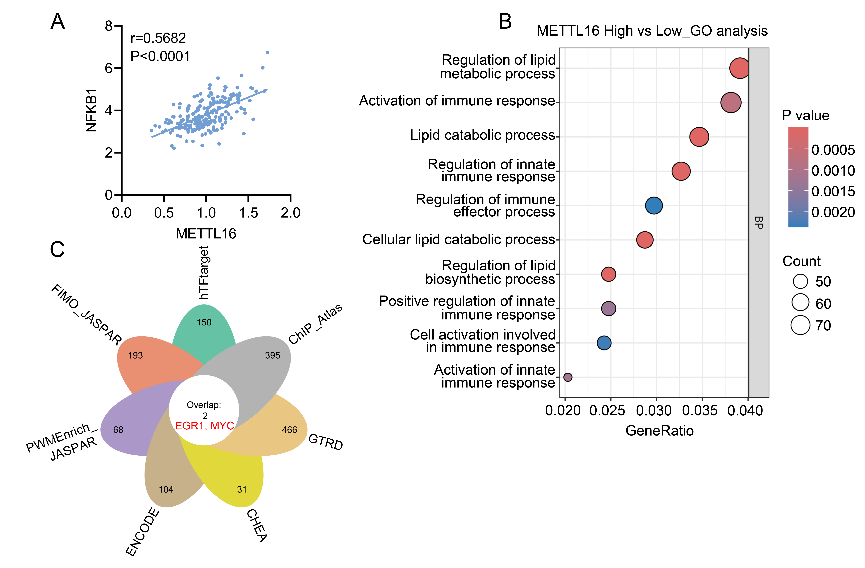
**Figure S4. Functional analysis and transcription factor prediction of METTL16.** (A) Correlation analysis between METTL16 and NFKB1 expression. (B) GO enrichment analysis of differentially expressed genes stratified by METTL16 expression level (high vs. low). (C) In silico prediction of candidate transcription factors regulating METTL16, using the ChIP-Atlas, GTRD, CHEA, ENCODE, PWMEnrich_JASPAR, FIMO_JASPAR, and hTFtarget databases.
